# Sorption of tetracycline on biochar derived from rice straw under different temperatures

**DOI:** 10.1371/journal.pone.0182776

**Published:** 2017-08-08

**Authors:** Hua Wang, Yixuan Chu, Chengran Fang, Fang Huang, Yali Song, Xiangdong Xue

**Affiliations:** 1 Key Laboratory of Recycling and Eco-treatment of Waste Biomass of Zhejiang Province, Zhejiang University of Science and Technology, Hangzhou, Zhejiang, China; 2 School of Civil Engineering and Architecture, Zhejiang University of Science and Technology, Hangzhou, Zhejiang, China; 3 Agricultural Comprehensive Inspection and Testing Center of Jiaxing, Jiaxing, Zhejiang, China; RMIT University, AUSTRALIA

## Abstract

Biochars produced from the pyrolysis of waste biomass under limited oxygen conditions could serve as adsorbents in environmental remediation processes. Biochar samples derived from rice straw that were pyrolyzed at 300 (R300), 500 (R500) and 700°C (R700) were used as adsorbents to remove tetracycline from an aqueous solution. Both the Langmuir and Freundlich models fitted the adsorption data well (R^2^ > 0.919). The adsorption capacity increased with pyrolysis temperature. The R500 and R700 samples exhibited relative high removal efficiencies across a range of initial tetracycline concentrations (0.5mg/L-32mg/L) with the maximum (92.8%–96.7%) found for adsorption on R700 at 35°C. The relatively high surface area of the R700 sample and π–π electron-donor acceptor contributed to the high adsorption capacities. A thermodynamic analysis indicated that the tetracycline adsorption process was spontaneous and endothermic. The pH of solution was also found to influence the adsorption processes; the maximum adsorption capacity occurred at a pH of 5.5. These experimental results highlight that biochar derived from rice straw is a promising candidate for low-cost removal of tetracycline from water.

## Introduction

Antibiotics are widely used to control infectious diseases and as animal feed additives to promote healthy growth. In China in 2013, annual antibiotics consumption was approximately 162,000 tons, with 52% of the total attributed to animal use [1]. Such levels of consumption notably increase the environmental concentration of antibiotics. This is because animals only adsorb a portion of the dosage given to them and a large volume of antibiotics is then excreted via urine and feces [2]. These residual antibiotics can then be taken up from the environment by plants and animals, and can even accumulate in the human food chain [3, 4]. In addition, the persistent levels of antibiotics in the environment promotes antibiotic resistance via gene transfer within, particularly pathogenic, bacteria [5]. Tetracycline is one of the most extensively used antibiotics and is often used in the livestock industry where wastewater analysis has shown tetracycline concentrations reaching tens of micrograms per liter [1, 6].

There are a number of methods available to combat antibiotic contamination of wastewater and the wider environment. These can generally be categorized as processes that employ advanced oxidation, electrochemistry, a biological agent, or a sorption system [7–9]. Adsorption is one of the most efficient means of limiting antibiotic contamination and displays advantages such as low cost, simple design and easy operation [10]. Adsorption processes have been used to remove around 30 types of antibiotics with removal efficiencies in the 90–100% range at mg/L concentration levels in contaminated waters [10]. Various adsorbents have been shown to be effective at removing tetracycline, including activated carbon (AC), carbon nanotubes and bentonite [11, 12]. However, these materials are expensive and lower cost alternatives are sought, particularly for large scale water treatment operations [13].

The pyrolysis of biomass yields biochar, which with its porous structure and medium-to-high surface area is similar to AC [14, 15]. The biochar surface contains a non-carbonized fraction that includes O-containing carboxyl, hydroxyl and phenolic functional groups, which can immobilize contaminants [16, 17]. Biochar sorbents have been shown to be an efficient and cost-effective means of removing antibiotic components including, tetracycline, sulfonamides, and ceftiofur from aqueous environments [18–21]. Liu et al. [21] reported the adsorption capacity for tetracycline of biochar derived from rice husks was 17 mg/g for batch experiments where tetracycline concentrations ranged from 50 to 1000 mg/L. However, the adsorption capacity of biochar depends on the biomass feedstock and the pyrolysis process because these factors influence biochar characteristics such as surface area, surface functional groups and porosity [19, 21, 22]. Other work has investigated the impact of the pH of the solution and the ionic strength of the biochar on antibiotics adsorption [21, 23]. However, little work assessing the adsorption of tetracycline on biochar produced under different temperature conditions has been reported.

Rice straw is one of the most abundant agricultural wastes in China [24]. Adopting this low cost, high carbon and abundant biochar feedstock could also decrease the amount that is currently burned, which is a contributor to local air pollution [25, 26]. Our objectives in this work are: 1) to investigate the characteristics of biochar derived from rice straw and its ability to adsorb tetracycline; 2) use adsorption isotherms and thermodynamic analysis to analyze the mechanism by which tetracycline and the biochar interact; and 3) study the effect of pH on the adsorption processes.

## Materials and methods

### Source materials

The rice straw was obtained from cropland in Hangzhou, Zhejiang province, China. Biochars were produced by pyrolyzing dried rice straw at three different temperatures (300, 500, and 700°C, yielding the R300, R500, and R700 samples, respectively). The pyrolysis temperature was raised at a rate of 26°C/min to the target temperature and then held for 2h. Once cool, the biochar samples were ground until they passed through a 0.15mm sieve.

The rice straw was the left over waste having no use for the farmers, and we have obtained permission from the farmer of this private cropland. We confirm that the field studies did not involve endangered or protected species.

The sorbate tetracycline (> 97.7% purity) was purchased from Dr Ehrenstorfer (Ausberg, Germany) while all other chemicals were analytical grade or better.

### Biochar characteristics

The biochar samples’ content of carbon (C), hydrogen (H), nitrogen (N) and oxygen (O) were analyzed in triplicate with an elemental analyzer (Vario MICRO, Elementar, Germany). The Brunauer–Emmett–Teller surface area (*S*_BET_), total pore volume (*V*_tot_), and pore size distribution of the biochar samples were analyzed in triplicate using N_2_ adsorption at 77 K with an Autosorb-IQC gas analyzer (Quantachrome, USA). Functional groups in the biochar samples were determined using Fourier transform infrared (FTIR) spectrometry (VERTEX 70, BRUKER, Germany).

### Adsorption experiments

The tetracycline adsorption experiments were carried out in a batch adsorption mode. A stock solution of tetracycline (100 mg/L) was prepared with Milli-Q water and methanol where the concentration of methanol was < 0.1% to minimize the potential co-solvent effects on sorption process [23]. Sorption isotherms were obtained across a tetracycline concentration range of 0.5 to 32 mg/L at temperatures of 15, 25 and 35°C. Solutions of 60 mg biochar and 20 ml tetracycline were added to 40-ml brown glass vials, which were fitted with a plastic cap. The vials were then shook at 150 rpm for 24 h in a constant temperature oscillator to reach an equilibrium state. Following being shaken, the samples were centrifuged at 5000 rpm for 10 min with the supernatant then filtered through a 0.45-μm membrane. The concentration of tetracycline in the supernatant was measured by high performance liquid chromatography (e2695, Waters, USA) using a C-18 column and a UV–vis spectrometer (2489, Waters, USA) operating at wavelength of 355 nm. A control sample (without biochar) was simultaneously prepared to assess any loss of tetracycline during the sorption process. However, no measurable change was observed for the tetracycline concentrations during the control experiment. All of the experiments were performed in triplicate.

The effect of changing the pH on the adsorption of tetracycline by biochar was determined in another series of batch experiments using a concentration of tetracycline of 32 mg/L. The solution pH was adjusted by using 0.1 mol/L HCl or NaOH to 2.0, 3.5, 5.5,7.5 and 10.0. A weighed amount of biochar (60 mg) was added to 20 mL tetracycline solution. The experiments were again performed in triplicate.

### Data analysis

The Langmuir (Eq (1)) and Freundlich (Eq (2)) adsorption models were used to fit the experimental data.

Langmuir:

$$q_{e}=q_{\max}K_{L}C_{e}/(1+K_{L}C_{e})$$ (1)

Freundlich:

$$q_{e}=K_{f}C_{e}^{n}$$ (2)

where *q*_e_ (mg/kg) is the rate of sorption of tetracycline on the biochar, *C*_e_ (mg/L) is the tetracycline concentration in the solution phase, *K*_*L*_ (L/mg) is the Langmuir sorption coefficient, *q*_max_ (mg/kg) is the sorption capacity, and *K*_*f*_ (mg^1−n^L^n^/kg) and *n* are the sorption and nonlinear coefficients in the Freundlich equation, respectively.

The efficiency with which a sample removed tetracycline (*R*) was calculated using Eq (3):

$$R=\frac{C_{0}-C_{e}}{C_{0}}\times 100\%$$ (3)

where *C*_0_ is the initial tetracycline concentration in the solution phase.

Eqs (4–6) were then used to calculate the change in the standard Gibbs free energy (Δ*G*), enthalpy (Δ*H*) and entropy (Δ*S*) [22]:

$$\Delta G=-RT\ln K_{d}$$ (4)

ΔG=ΔH−TΔS (5)

$$\ln K_{d}=-\Delta H/RT+\Delta S/R$$ (6)

where *K*_d_ = *q*_e_/*C*_e_ and represents the sorption coefficient, *T* (K) is the absolute temperature and *R* (8.314 J mol^−1^ K^−1^) is the universal gas constant. When ln*K*_d_ is plotted against 1/*T*, a straight line with a slope of -Δ*H*/*R* and an intercept of Δ*S*/*R* is obtained.

Statistical analysis was performed by SPSS 16.0 (Chicago, IL). For Δ*G* values, a two-way ANOVA was applied to examine the effects of pyrolysis and adsorption temperature, the significant differences among treatments were compared with the Tukey’s test at each initial tetracycline concentration (S1 Table). For Δ*S* and Δ*H* values, a one-way ANOVA was applied to examine the effects of pyrolysis temperature, the significant differences were also tested using Tukey’s test (S1 Table). In order to investigate how chemical properties and pore structure of biochars influence the biochar sorption, correlation analysis (n = 9) was used to examine the relationship between biochar characteristics and the *q*_e_ values obtained at each initial tetracycline concentration at 25°C.

## Results and discussion

### Biochar characteristics

The chemical properties of the biochar samples derived from rice-straw are shown in Table 1. The O and H content of the samples decreased rapidly as the pyrolysis temperature was increased. Meanwhile, the C content increased from 45.2% for a pyrolysis temperature of 300°C to 56.0% when the temperature was 700°C. The ratios of O/C, H/C and (O+N)/C exhibited a similar trend to that of the biochar’s O content. All of these findings were consistent with those reported in previous studies [27]. The fact that lower O/C ratios were observed for samples that were pyrolyzed at higher temperatures indicated that these biochars likely had less hydrophilic surfaces [16]. Taken together, these changes in the chemical composition of the biochar samples indicated that the higher pyrolysis temperatures promoted a higher degree of carbonization.

**Table 1:** Chemical composition of biochars derived from rice straw.

Biochar	C*(%)	N(%)	O(%)	H(%)	O/C	H/C	(O+N)/C
R300	45.22±0.46	1.47±0.06	23.41±0.35	4.94±0.13	0.52	0.11	0.55
R500	52.60±1.06	1.35±0.04	15.57±0.24	2.15±0.07	0.30	0.04	0.32
R700	56.00±0.87	1.18±0.04	11.38±0.10	1.29±0.09	0.20	0.02	0.22

*The content of C, N, O and H elements in the biochar.

**Fig 1:**
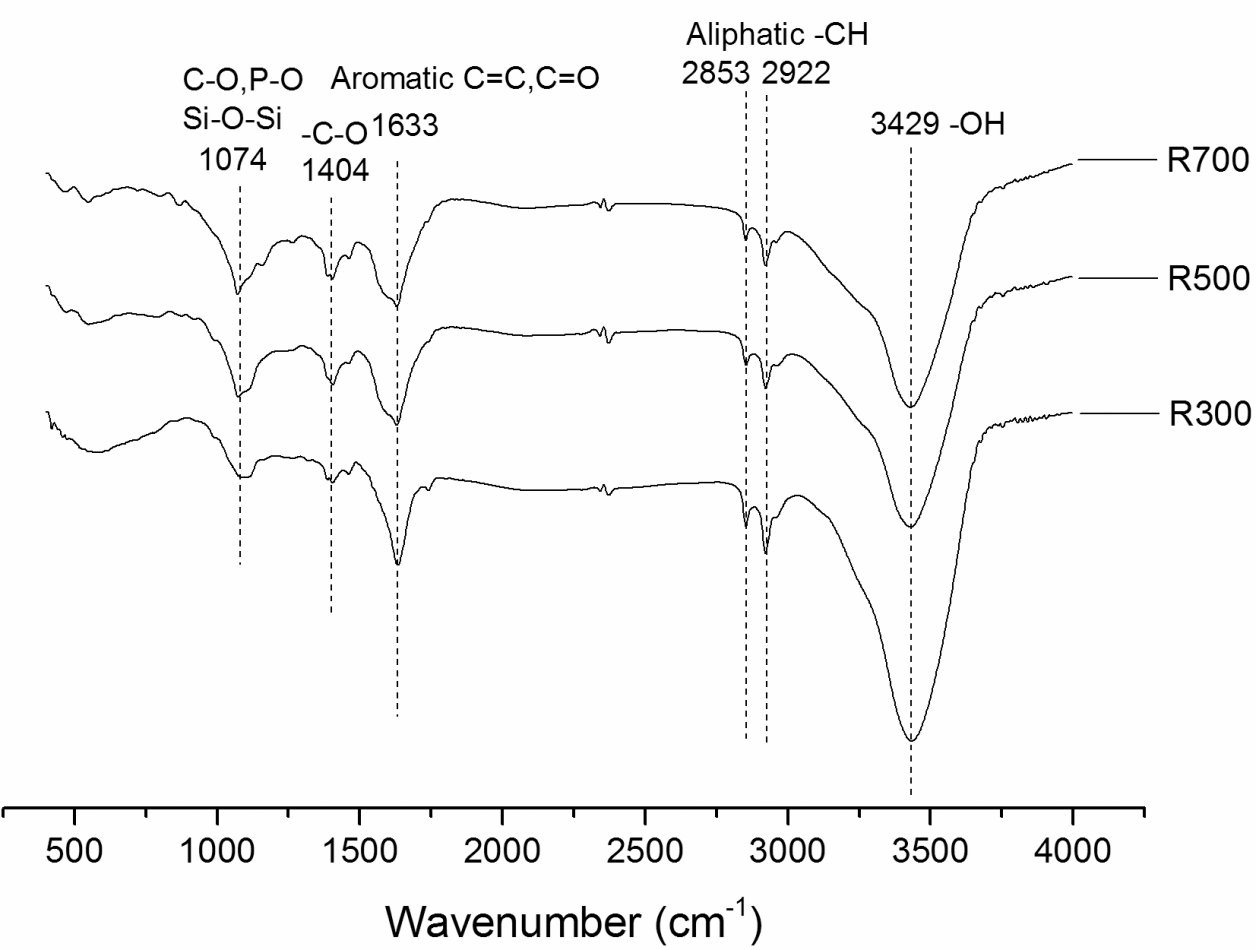
Fourier transform infrared (FTIR) spectroscopic analysis of biochars derived from rice straw.

The surface area (*S*_BET_) of the biochar samples increased dramatically with temperature (from 2.48 m^2^/g in R300 to 27.66 m^2^/g in R700), showing the same trend as the pore volume (as shown in Table 2), both of which were in agreement with previous work [22]. From the FTIR analysis (Fig 1), the peaks at 2922 and 2853 cm^−1^ reflected the presence of aliphatic–CH, while the absorption at 3429 cm^−1^ was assigned to surface–OH groups [21]. The peaks at these three wavenumbers were smaller for the biochar pyrolyzed at 700°C, which indicated that increasing the pyrolysis temperature decreased the content of O-containing and aliphatic carbon functional groups. Adsorptions in the region 1000 to 1100 cm^−1^ were ascribed to Si–O–Si groups, the P–O bond of phosphate and the C–O bond of carbonate [28]. All of these showed the strongest adsorptions in the R700 samples, indicating the highest mineral salt content occurred in the sample with the highest pyrolysis temperature.

**Table 2:** Pore structure of biochars derived from rice straw.

Biochar	*S*_BET_^a^ (m^2^/g)	*V*_tot_ (cm^3^/g)	Pore width (nm)
R300	2.48±0.84	0.048±0.008	17.06±1.01
R500	10.18±1.52	0.057±0.011	15.27±1.20
R700	27.66±4.64	0.059±0.010	21.55±2.58

^a^The *S*_BET_ and *V*_tot_ represent the Brunauer–Emmett–Teller surface area and total pore volume respectively.

### Tetracycline sorption

**Fig 2:**
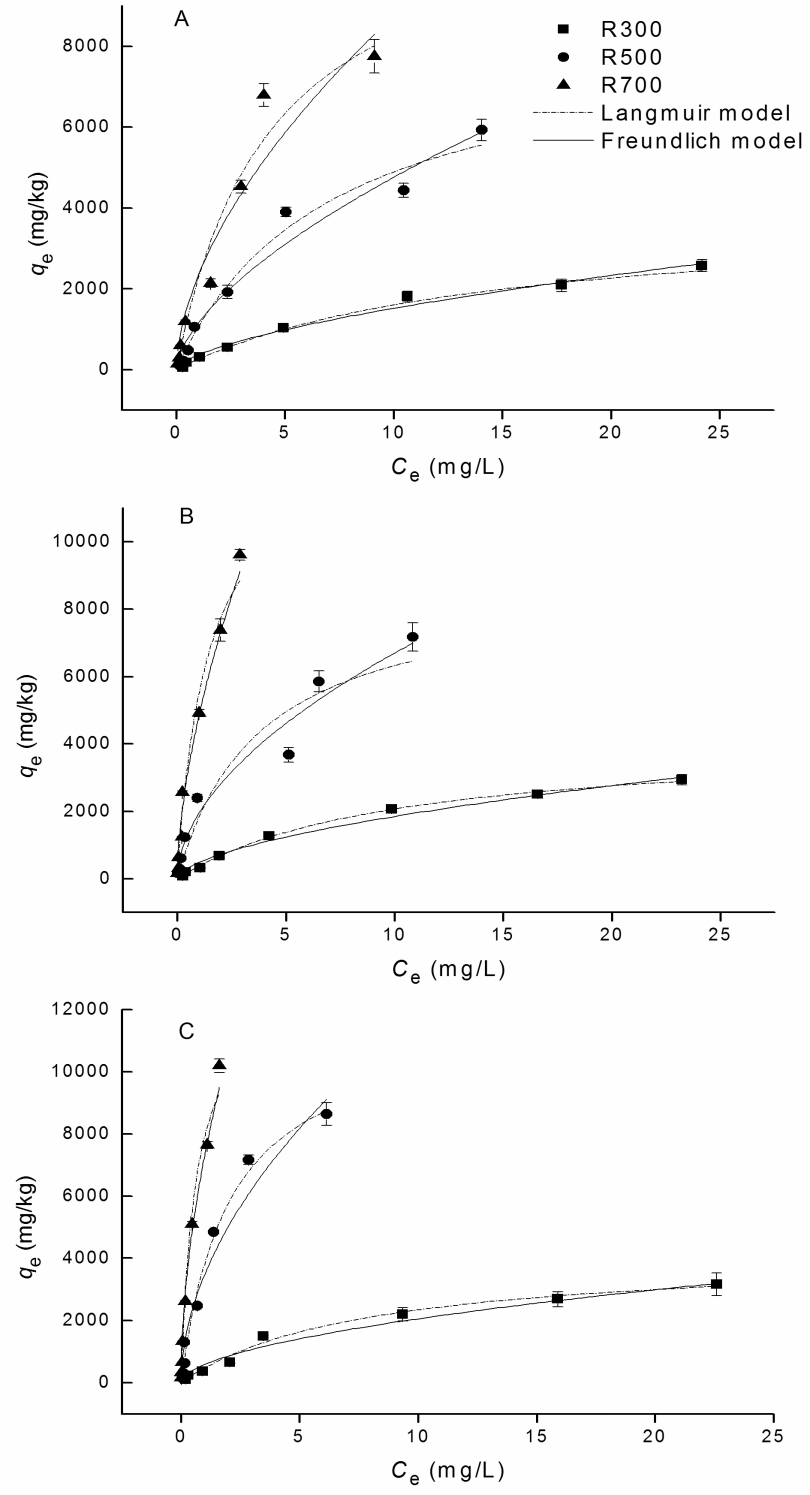
Sorption isotherms for tetracycline on biochars. The adsorption temperatures were (A) 15°C, (B) 25°C and (C) 35°C.

The sorption isotherms of tetracycline on the biochar samples are shown in Fig 2. The adsorption data showed a good fit to both the Langmuir and the Freundlich models with R^2^ coefficients higher than 0.919 in the experimental concentration ranges (as shown in Table 3). For the parameters in the Freundlich model, the values of *n* for the different samples were similar (0.53–0.62) and less than 1, which indicated that the adsorption was a concentration dependent process. The value of *K*_f_, which reflects a sample’s ability to absorb tetracycline, was highest in the biochar produced by the 700°C pyrolysis. The trend shown by the *q*_max_ values in Langmuir model also suggested that sorption capacity increased with pyrolysis temperature. The maximum adsorption capacities were calculated to lie in the approximate range of 11,834–14,157 mg/kg.

**Table 3:** Langmuir and Freundlich model parameters for tetracycline sorption on biochars derived from rice straw at different adsorption temperatures (T).

Bioc-har	T(°C)	Freundlich model			Langmiur model		
		*K*_f_(10^3^mg^1-n^L^n^/kg)	n	R^2^	*q*_max_(10^3^mg/kg)	*K*_L_(L/mg)	R^2^
R300	15	0.364±0.034	0.62±0.03	0.975	3.889±0.244	0.07±0.01	0.984
	25	0.486±0.040	0.58±0.03	0.976	4.136±0.158	0.10±0.01	0.992
	35	0.593±0.068	0.54±0.04	0.947	4.147±0.282	0.13±0.02	0.965
R500	15	1.141±0.121	0.62±0.05	0.953	8.384±0.734	0.14±0.03	0.966
	25	1.930±0.120	0.54±0.05	0.937	8.756±1.159	0.26±0.10	0.925
	35	3.484±0.234	0.53±0.04	0.938	11.695±0.730	0.49±0.07	0.952
R700	15	2.301±0.249	0.58±0.06	0.919	11.834±1.417	0.23±0.06	0.938
	25	4.817±0.289	0.60±0.06	0.931	14.157±1.522	0.71±0.18	0.946
	35	7.307±0.333	0.54±0.05	0.930	13.849±1.359	1.61±0.42	0.942

Surface area is known to be an important factor that influences the adsorption capacities of biochar, and specifically in the case of tetracycline removal [21, 29]. This was confirmed in this study, the surface area (*S*_BET_) of the biochar samples was significantly positively correlated with *q*_e_ values (Table 4). The adsorption capacities increased as the biochar samples’ surface area increased following exposure to higher pyrolysis temperatures.

**Table 4:** Pearson’s linear correlation coefficients between biochar characteristics and the *q*_e_ values obtained at different initial tetracycline concentration(*C*_0_) at 25°C (n = 9).

*C*_0_(mg/L)	C^a^	N	O	H	O/C	H/C	(O+N)/C	*S*_BET_^b^	Vtot	Pore width
0.5	0.925**	-0.751**	-0.923**	-0.962**	-0.944**	-0.969**	-0.943**	0.690*	0.655	0.186
1.0	0.954**	-0.849**	-0.978**	-0.974**	-0.979**	-0.971**	-0.979**	0.843**	0.701*	0.439
2.0	0.965**	-0.802**	-0.957**	-0.984**	-0.974**	-0.990**	-0.973**	0.758*	0.656	0.280
4.0	0.938**	-0.783**	-0.933**	-0.973**	-0.952**	-0.979**	-0.952**	0.717*	0.648	0.225
8.0	0.957**	-0.844**	-0.969**	-0.988**	-0.979**	-0.990**	-0.978**	0.793*	0.676*	0.340
16.0	0.962**	-0.948**	-0.989**	-0.971**	-0.980**	-0.962**	-0.981**	0.941**	0.669	0.609
24.0	0.987**	-0.899**	-0.993**	-0.986**	-0.994**	-0.983**	-0.994**	0.876**	0.627	0.473
32.0	0.977**	-0.929**	-0.997**	-0.985**	-0.993**	-0.979**	-0.993**	0.902**	0.648	0.530

*Correlation is significant at the 0.05 level (2-tailed).

**Correlation is significant at the 0.01 level (2-tailed).

^a^ The content of C, N, O and H elements in the biochar.

^b^The *S*_BET_ and *V*_tot_ represent the Brunauer–Emmett–Teller surface area and total pore volume respectively.

The properties of the antibiotic and the biochar affect the adsorption process and the various underlying mechanisms, which include electrostatic, hydrophobic, hydrogen bond and π–π electron-donor acceptor (EDA) interactions [19, 30]. Previous studies have shown that electrostatic interaction (cation and anion attractions) and hydrophobic interaction do not dominate the adsorption of tetracycline on biochar [19, 21]. Under the conditions in this study (pH = 5.5), tetracycline presented as a zwitterion. Thus, although the net charge of tetracycline was neutral, the negative and positive charges were spatially separated within the tetracycline molecule, and acted independently during the adsorption processes [31]. The pH of the isoelectric point of the biochar samples inferred from previous study was lower than that of the solution [32], suggesting that the surface of the biochar samples was negatively charged. Therefore, it is unlikely that hydrophobic interaction or electrostatic interaction (cation and anion attractions) played a dominant role in the adsorption process in the current work. The FTIR analysis indicated that R300 possessed more O-containing functional groups, which can serve as H-bond acceptors. However, the fact that R300’s adsorption capacity was the lowest of all of the samples which suggested that hydrogen bond interactions also did not play a dominant role in the adsorption process.

Conversely, EDA interactions have been reported to be one of the main mechanisms controlling biochar’s adsorption of tetracycline and other molecules [19, 23, 33]. The biochar samples in this work possessed a graphite-like structure which can act as a π-electron-donor [11]. Meanwhile, tetracycline can serve as an π-electron-acceptors thanks to its aromatic ring structure. In this study, *q*_e_ values were positively correlated with the content of C in the biochar (Table 4). As the pyrolysis temperature was increased, the degree of graphitization of biochar increased, thus enhancing the sample’s adsorption capacity.

### Tetracycline adsorption capacity and removal efficiency

**Fig 3:**
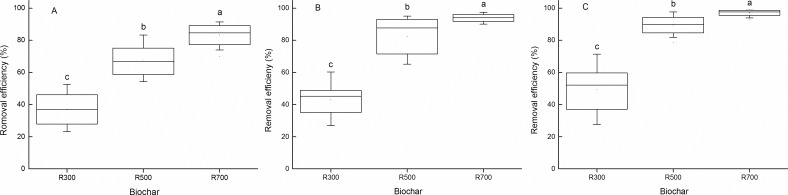
Removal efficiency of tetracycline by biochars. The adsorption temperatures were (A) 15°C, (B) 25°C and (C) 35°C. The initial tetracycline concentration changed from 0.5 mg/L to 32 mg/L.

The calculated efficiencies with which the biochar samples removed tetracycline are presented in Fig 3. The R500 and R700 samples showed relatively high removal efficiencies with the highest (92.8–96.7%) calculated for the R700 sample for adsorption at 35°C. The tetracycline adsorption capacity of various materials has been reported to follow the following order: carbon nanotubes > AC > bentonite [10, 11, 34]. The adsorption capacities of the samples in this work (which maximum 14.16 mg/g) were lower than those reported for AC (typically 100–1000 mg/g) [11, 35], but higher than those of clay, soil and rice husk ash [13, 36, 37]. The removal efficiencies, particularly those of the samples pyrolyzed at higher temperatures, were similar to those observed for AC (74–88% for a tetracycline concentration of 20 mg/L) [34]. Taken together, the results suggest that biochar derived from rice straw appears to be a useful means of treating tetracycline-contaminated water.

### Thermodynamics analysis

**Fig 4:**
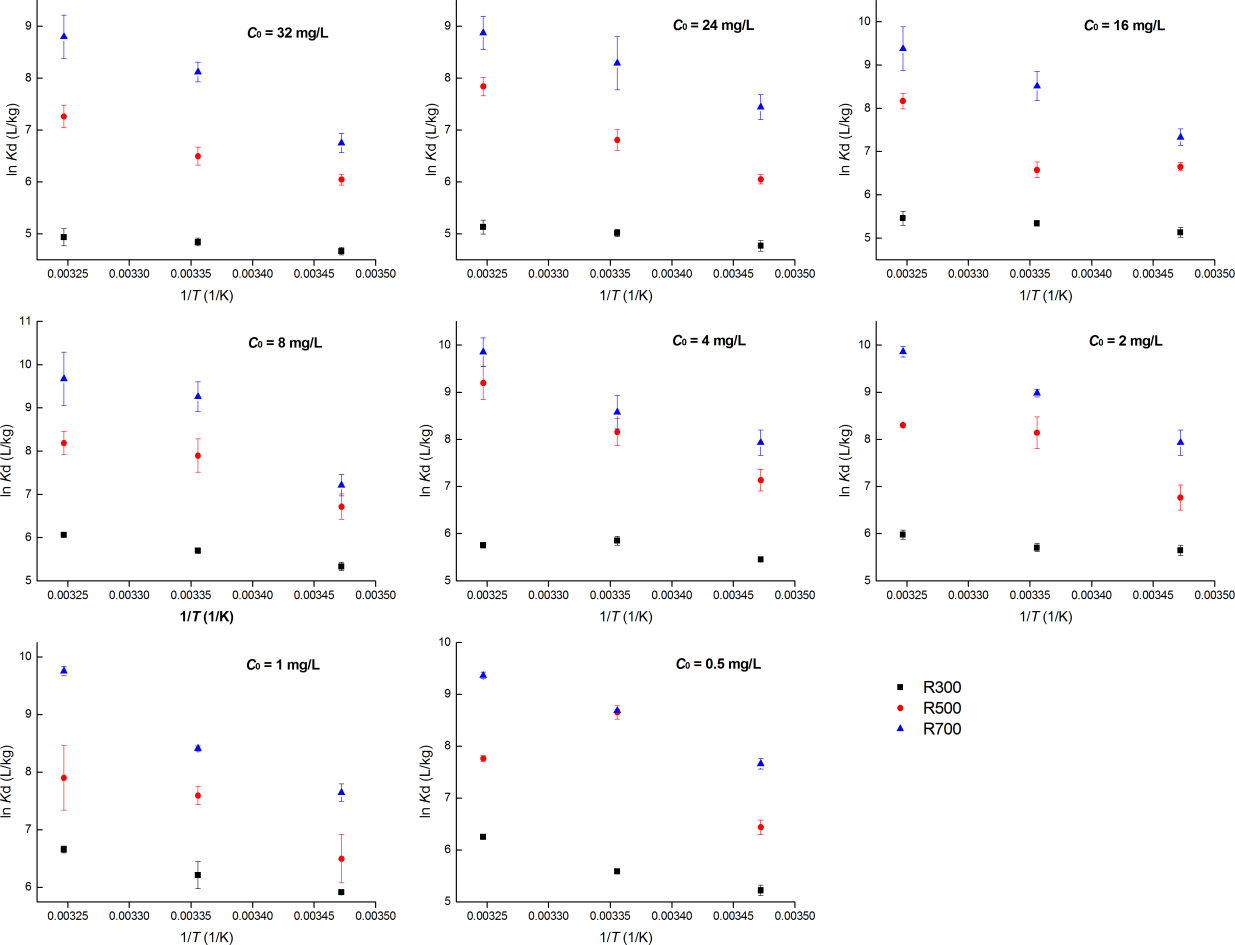
Effect of temperature on sorption coefficient (*K*_d_) for tetracycline sorption on biochars with different initial tetracycline concentration.

Analysis of the thermodynamics of the sorption process can help to illustrate the underlying mechanisms and the impact on them of the adsorption temperature. Fig 4 showed the relationship between ln*K*_d_ and 1/*T*. The Δ*G* values calculated from ln*K*_d_. Negative Δ*G* values were detected for all samples (S1 Table, Table 5). This suggested, in agreement with previous studies [13, 38], that the sorption of tetracycline on biochar derived from rice straw was thermodynamically favorable and spontaneous for all of the pyrolysis temperatures investigated. The increase in the magnitude of Δ*G* with the temperature of the sorption environment (S1 Table, Table 5) indicated that the tetracycline sorption process was more thermodynamically favorable at higher sorption temperatures. Unlike the sorption of sulfamethoxazole on biochar [22], the magnitude of Δ*G* increased with the pyrolysis temperature, again suggesting that increased temperature (this time during the biochar preparation) yielded a more thermodynamically favorable sorption process. At same time, the Δ*G* values of adsorption processes in this work were higher than those reported for graphene oxide (from -3.27 kJ/mol to -2.01 kJ/mol) [39]and multi-walled carbon nanotubes (from -5.54 kJ/mol to -3.99 kJ/mol) [40] but lower than those of activated carbon nanoparticles (from -87.97 kJ/mol to -82.60 kJ/mol) [34].

**Table 5:** Thermodynamic parameters for tetracycline sorption on biochars with initial concentration of tetracycline changing from 0.5 mg/L to 32 mg/L.

Biochar	Temperature(°C)	Thermodynamic parameters		
		Δ*G* (kJ/mol)	Δ*H* (kJ/mol)	Δ*S* (J/(mol·K))
R300	15	-14.17–-11.17^a^	11.05–37.76	81.85–174.06
	25	-15.40–-11.99		
	35	-17.05–-12.64		
R500	15	-17.08–-14.47	44.64–76.10	204.80–323.43
	25	-21.43–-16.10		
	35	-23.55–-18.59		
R700	15	-18.99–-16.16	52.85–91.27	282.14–378.80
	25	-22.94–-20.11		
	35	-25.24–-22.52		

^a^Minimum–Maximum.

The Δ*H* values for the various samples ranged from 11.05 to 91.27 kJ/mol (S1 Table, Table 5) indicating that the overall process was endothermic. This finding also suggested that the adsorption process was more favorable at higher temperatures, which is consistent with the trend in adsorption capacities described in Table 2. This result may be due to the increase in diffusion rate of tetracycline as the temperature increases. An increased number of tetracycline molecules may also acquire sufficient energy to interact with active sites on the surface of the biochar [41].The trend in Δ*S* was similar to that for Δ*H*, with the positive Δ*S* values revealing that the adsorption process was favored sorption stability [42]. As Δ*S* increased with the pyrolysis temperature (S1 Table), this indicated randomness increased at the tetracycline–biochar interface during the adsorption process [13]. The net positive entropy may be attributed to that the positive entropy change caused by the changes in the surface of the biochar exceeds numerically the negative entropy change result from loss of freedom of the tetracycline [43]. Compared to some other carbon-material, the Δ*H* and Δ*S* values in this study were lower than those reported for activated carbon nonoparticles [34] but higher than those reported for multi-walled carbon nanotubes [40]. As to graphene oxide, only the R700 exhibit higher Δ*H* and Δ*S* values compared to graphene oxide [39].

### The impact of pH

**Fig 5:**
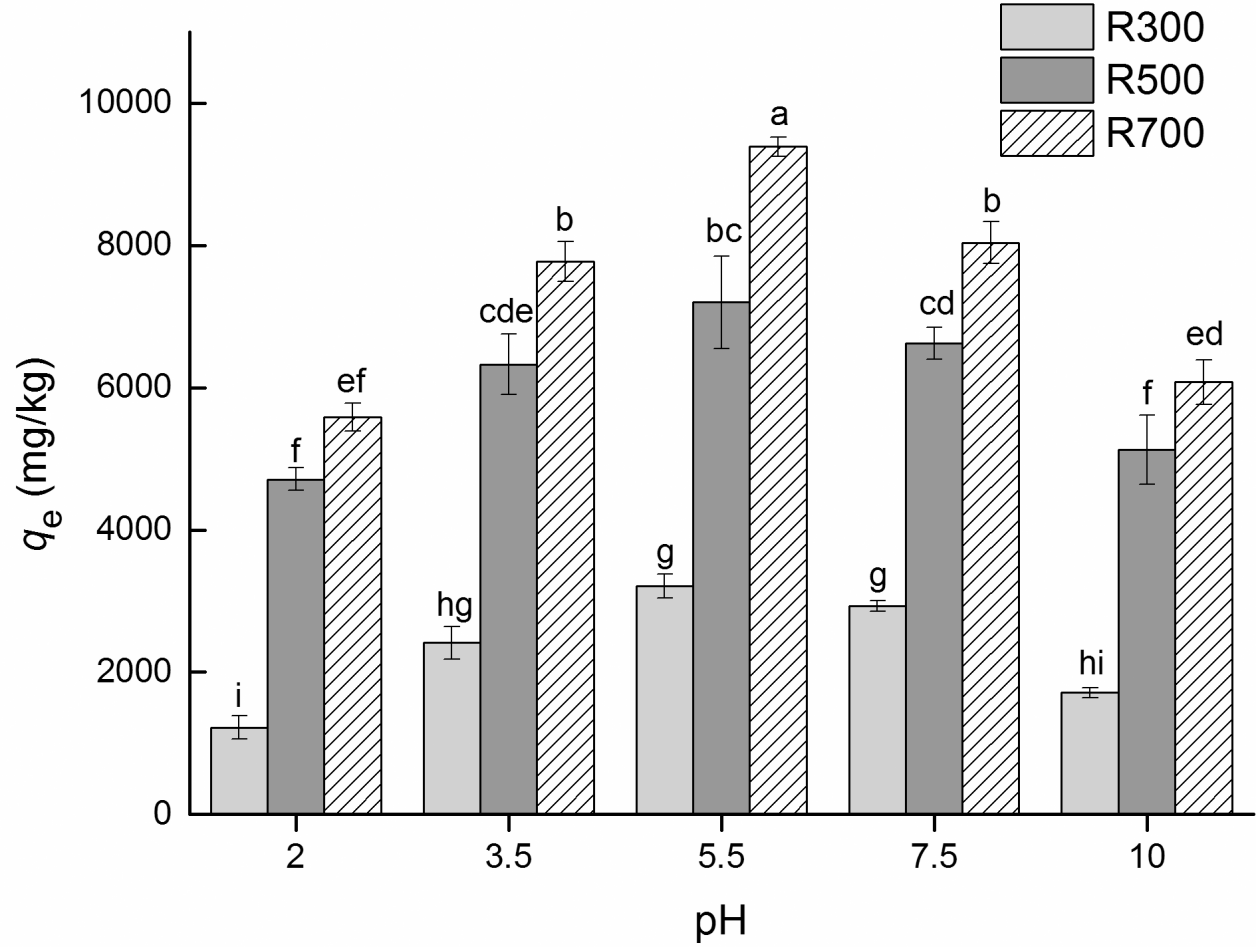
Effect of pH on adsorption capacity for tetracycline sorption on biochars. The concentration of tetracycline solution was 32 mg/L. Values are triplicate means ± SD. Letters indicate significant differences among treatments at a level of p < 0.05(Tukey test).

The adsorption capacity for different biochar samples under various pH conditions are shown in Fig 5. For all pH conditions, tetracycline adsorption capacity increased with pyrolysis temperature. For all samples, as pH was increased, the samples’ adsorption capacity initially increased but then fell, which was consistent with previous studies [23]. The pH of the solution influenced the adsorption processes by changing the surface charges of the biochar and tetracycline [21, 23]. tetracycline is amphoteric and depending on the pH can be a cation (pH < 3.3), a zwitterion (3.3 < pH < 7.7) or an anion (pH > 7.7) [42]. Previous studies evaluated the isoelectric point of biochar derived from rice straw to be between 3 and 4 [32, 44]. Thus, when the solution pH was 2, the biochar surface was positively charged. However, increasing the pH caused the biochar’s surface to become negatively charged. Electrostatic repulsion occurred when the pH was outside of the range of 3.3–7.7, which resulted in decreased adsorption capacities at these pH values.

## Conclusions

This study showed the biochars pyrolyzed at 500 and 700°C were relatively high efficient at removing tetracycline from an aqueous solution. The high adsorption capacity for the R700 sample was attributed to the relatively high surface area and EDA interactions between the tetracycline molecules and the biochar’s graphitic surfaces. A thermodynamic analysis indicated that adsorption of tetracycline on biochar was thermodynamically favorable and spontaneous, and that Δ*H* and Δ*S* increased with pyrolysis temperature. The pH of the solution also influenced the adsorption processes with a maximum adsorption capacity occurring at pH = 5.5. Overall, biochar derived by pyrolyzing rice straw offers a promising option for a low-cost method of removing tetracycline from water and could be used to mitigate tetracycline pollution of wastewater from livestock farms. However, it is important to clarify that this study was based on aqueous solution, the matrix of wastewater is relatively complex and so the practical application need further investigation.

## Supporting information

S1 Table Thermodynamic parameters for tetracycline sorption on biochars at different initial concentration of tetracycline (C_0_). (DOC)

## References

[1] Zhang Q-Q, Ying G-G, Pan C-G, Liu Y-S, Zhao J-L. Comprehensive evaluation of antibiotics emission and fate in the river basins of China: source analysis, multimedia modeling, and linkage to bacterial resistance. Environ Sci Technol. 2015;49(11):6772–82. doi: 10.1021/acs.est.5b00729 25961663 10.1021/acs.est.5b00729

[2] Zhao L, Dong YH, Wang H. Residues of veterinary antibiotics in manures from feedlot livestock in eight provinces of China. Sci Total Environ. 2010;408(5):1069–75. doi: 10.1016/j.scitotenv.2009.11.014 19954821 10.1016/j.scitotenv.2009.11.014

[3] Cabello FC. Heavy use of prophylactic antibiotics in aquaculture: a growing problem for human and animal health and for the environment. Environ Microbiol. 2006;8(7):1137–44. doi: 10.1111/j.1462-2920.2006.01054.x 16817922 10.1111/j.1462-2920.2006.01054.x

[4] Kumar K, Gupta S, Baidoo S, Chander Y, Rosen C. Antibiotic uptake by plants from soil fertilized with animal manure. J Environ Qual. 2005;34(6):2082–5. doi: 10.2134/jeq2005.0026 16221828 10.2134/jeq2005.0026

[5] Knapp CW, Dolfing J, Ehlert PA, Graham DW. Evidence of increasing antibiotic resistance gene abundances in archived soils since 1940. Environ Sci Technol. 2009;44(2):580–7. doi: 10.1021/es901221x 20025282 10.1021/es901221x

[6] Chen Y, Zhang H, Luo Y, Song J. Occurrence and dissipation of veterinary antibiotics in two typical swine wastewater treatment systems in east China. Environ Monit Assess. 2012;184(4):2205–17. doi: 10.1007/s10661-011-2110-y 21633797 10.1007/s10661-011-2110-y

[7] Jeong J, Song W, Cooper WJ, Jung J, Greaves J. Degradation of tetracycline antibiotics: mechanisms and kinetic studies for advanced oxidation/reduction processes. Chemosphere. 2010;78(5):533–40. doi: 10.1016/j.chemosphere.2009.11.024 20022625 10.1016/j.chemosphere.2009.11.024

[8] Oller I, Malato S, Sánchez-Pérez J. Combination of advanced oxidation processes and biological treatments for wastewater decontamination—a review. Sci Total Environ. 2011;409(20):4141–66. doi: 10.1016/j.scitotenv.2010.08.061 20956012 10.1016/j.scitotenv.2010.08.061

[9] Dirany A, Sirés I, Oturan N, Özcan A, Oturan MA. Electrochemical treatment of the antibiotic sulfachloropyridazine: kinetics, reaction pathways, and toxicity evolution. Environ Sci Technol. 2012;46(7):4074–82. doi: 10.1021/es204621q 22332959 10.1021/es204621q

[10] Ahmed MB, Zhou JL, Ngo HH, Guo W. Adsorptive removal of antibiotics from water and wastewater: progress and challenges. Sci Total Environ. 2015;532:112–26. doi: 10.1016/j.scitotenv.2015.05.130 26057999 10.1016/j.scitotenv.2015.05.130

[11] Ji L, Chen W, Duan L, Zhu D. Mechanisms for strong adsorption of tetracycline to carbon nanotubes: a comparative study using activated carbon and graphite as adsorbents. Environ Sci Technol. 2009;43(7):2322–7. doi: 10.1021/es803268b 19452881 10.1021/es803268b

[12] Rivera-Utrilla J, Gómez-Pacheco CV, Sánchez-Polo M, López-Peñalver JJ, Ocampo-Pérez R. Tetracycline removal from water by adsorption/bioadsorption on activated carbons and sludge-derived adsorbents. J Environ Manage. 2013;131:16–24. doi: 10.1016/j.jenvman.2013.09.024 24140483 10.1016/j.jenvman.2013.09.024

[13] Chen Y, Wang F, Duan L, Yang H, Gao J. Tetracycline adsorption onto rice husk ash, an agricultural waste: Its kinetic and thermodynamic studies. J Mol Liq. 2016;222:487–94. doi: 10.1016/j.molliq.2016.07.090

[14] Cao X, Ma L, Liang Y, Gao B, Harris W. Simultaneous immobilization of lead and atrazine in contaminated soils using dairy-manure biochar. Environ Sci Technol. 2011;45(11):4884–9. doi: 10.1021/es103752u 21542567 10.1021/es103752u

[15] Lehmann J, Joseph S. Biochar for environmental management: an introduction In: Lehmann J, Joseph S, editors. Biochar for environmental management: science, technology and implementation. New York: Routledge; 2015 p. 1–14.

[16] Chun Y, Sheng G, Chiou CT, Xing B. Compositions and sorptive properties of crop residue-derived chars. Environ Sci Technol. 2004;38(17):4649–55. doi: 10.1021/es035034w 15461175 10.1021/es035034w

[17] Ahmad M, Rajapaksha AU, Lim JE, Zhang M, Bolan N, Mohan D, et al Biochar as a sorbent for contaminant management in soil and water: a review. Chemosphere. 2014;99:19–33. doi: 10.1016/j.chemosphere.2013.10.071 24289982 10.1016/j.chemosphere.2013.10.071

[18] Shimabuku KK, Kearns JP, Martinez JE, Mahoney RB, Moreno-Vasquez L, Summers RS. Biochar sorbents for sulfamethoxazole removal from surface water, stormwater, and wastewater effluent. Water Res. 2016;96:236–45. doi: 10.1016/j.watres.2016.03.049 27060527 10.1016/j.watres.2016.03.049

[19] Liao P, Zhan Z, Dai J, Wu X, Zhang W, Wang K, et al Adsorption of tetracycline and chloramphenicol in aqueous solutions by bamboo charcoal: A batch and fixed-bed column study. Chem Eng J. 2013;228:496–505. doi: 10.1016/j.cej.2013.04.118

[20] Mitchell SM, Subbiah M, Ullman JL, Frear C, Call DR. Evaluation of 27 different biochars for potential sequestration of antibiotic residues in food animal production environments. J Environ Chem Eng. 2015;3(1):162–9. doi: 10.1016/j.jece.2014.11.012

[21] Liu P, Liu W-J, Jiang H, Chen J-J, Li W-W, Yu H-Q. Modification of bio-char derived from fast pyrolysis of biomass and its application in removal of tetracycline from aqueous solution. Bioresour Technol. 2012;121:235–40. doi: 10.1016/j.biortech.2012.06.085 22858491 10.1016/j.biortech.2012.06.085

[22] Lian F, Sun B, Song Z, Zhu L, Qi X, Xing B. Physicochemical properties of herb-residue biochar and its sorption to ionizable antibiotic sulfamethoxazole. Chem Eng J. 2014;248:128–34. doi: 10.1016/j.cej.2014.03.021

[23] Jia M, Wang F, Bian Y, Jin X, Song Y, Kengara FO, et al Effects of pH and metal ions on oxytetracycline sorption to maize-straw-derived biochar. Bioresour Technol. 2013;136:87–93. doi: 10.1016/j.biortech.2013.02.098 23567668 10.1016/j.biortech.2013.02.098

[24] Lei Z, Chen J, Zhang Z, Sugiura N. Methane production from rice straw with acclimated anaerobic sludge: effect of phosphate supplementation. Bioresour Technol. 2010;101(12):4343–8. doi: 10.1016/j.biortech.2010.01.083 20153179 10.1016/j.biortech.2010.01.083

[25] Zhang H, Hu D, Chen J, Ye X, Wang SX, Hao JM, et al Particle size distribution and polycyclic aromatic hydrocarbons emissions from agricultural crop residue burning. Environ Sci Technol. 2011;45(13):5477–82. doi: 10.1021/es1037904 21615081 10.1021/es1037904

[26] Sun J, Lian F, Liu Z, Zhu L, Song Z. Biochars derived from various crop straws: characterization and Cd (II) removal potential. Ecotox Environ Safe. 2014;106:226–31. doi: 10.1016/j.ecoenv.2014.04.042 24859708 10.1016/j.ecoenv.2014.04.042

[27] Gai X, Wang H, Liu J, Zhai L, Liu S, Ren T, et al Effects of feedstock and pyrolysis temperature on biochar adsorption of ammonium and nitrate. PLos One. 2014;9(12):e113888 doi: 10.1371/journal.pone.0113888 25469875 10.1371/journal.pone.0113888 PMC4254611

[28] Dai Z, Meng J, Shi Q, Xu B, Lian Z, Brookes P, et al Effects of manure‐and lignocellulose‐derived biochars on adsorption and desorption of zinc by acidic types of soil with different properties. Eur J Soil Sci. 2016;67(1):40–50. doi: 10.1111/ejss.12290

[29] Zhu X, Liu Y, Zhou C, Luo G, Zhang S, Chen J. A novel porous carbon derived from hydrothermal carbon for efficient adsorption of tetracycline. Carbon. 2014;77:627–36. doi: 10.1016/j.carbon.2014.05.067

[30] Tan X, Liu Y, Zeng G, Wang X, Hu X, Gu Y, et al Application of biochar for the removal of pollutants from aqueous solutions. Chemosphere. 2015;125:70–85. doi: 10.1016/j.chemosphere.2014.12.058 25618190 10.1016/j.chemosphere.2014.12.058

[31] Sassman SA, Lee LS. Sorption of three tetracyclines by several soils: assessing the role of pH and cation exchange. Environ Sci Technol. 2005;39(19):7452–9. doi: 10.1021/es0480217 16245815 10.1021/es0480217

[32] Qian L, Chen B. Interactions of aluminum with biochars and oxidized biochars: Implications for the biochar aging process. J Agric Food Chem. 2014;62(2):373–80. doi: 10.1021/jf404624h 24364719 10.1021/jf404624h

[33] Xie M, Chen W, Xu Z, Zheng S, Zhu D. Adsorption of sulfonamides to demineralized pine wood biochars prepared under different thermochemical conditions. Environ Pollut. 2014;186:187–94. doi: 10.1016/j.envpol.2013.11.022 24384578 10.1016/j.envpol.2013.11.022

[34] Pouretedal H, Sadegh N. Effective removal of amoxicillin, cephalexin, tetracycline and penicillin G from aqueous solutions using activated carbon nanoparticles prepared from vine wood. J Water Process Eng. 2014;1:64–73. doi: 10.1016/j.jwpe.2014.03.006

[35] Sayğılı H, Güzel F. Effective removal of tetracycline from aqueous solution using activated carbon prepared from tomato (Lycopersicon esculentum Mill.) industrial processing waste. Ecotox Environ Safe. 2016;131:22–9. doi: 10.1016/j.ecoenv.2016.05.001 27177317 10.1016/j.ecoenv.2016.05.001

[36] Pils JR, Laird DA. Sorption of tetracycline and chlortetracycline on K-and Ca-saturated soil clays, humic substances, and clay-humic complexes. Environ Sci Technol. 2007;41(6):1928–33. doi: 10.1021/es062316y 17410786 10.1021/es062316y

[37] Wang Y-J, Sun R-J, Xiao A-Y, Wang S-Q, Zhou D-M. Phosphate affects the adsorption of tetracycline on two soils with different characteristics. Geoderma. 2010;156(3):237–42. doi: 10.1016/j.geoderma.2010.02.022

[38] Chao Y, Zhu W, Yan B, Lin Y, Xun S, Ji H, et al Macroporous polystyrene resins as adsorbents for the removal of tetracycline antibiotics from an aquatic environment. J Appl Polym Sci. 2014;131(15):1–8. doi: 10.1002/app.40561

[39] Ghadim EE, Manouchehri F, Soleimani G, Hosseini H, Kimiagar S, Nafisi S. Adsorption properties of tetracycline onto graphene oxide: equilibrium, kinetic and thermodynamic studies. Plos One. 2013;8(11):e79254 doi: 10.1371/journal.pone.0079254 24302989 10.1371/journal.pone.0079254 PMC3841164

[40] Zhang L, Song X, Liu X, Yang L, Pan F, Lv J. Studies on the removal of tetracycline by multi-walled carbon nanotubes. Chem Eng J. 2011;178:26–33. doi: 10.1016/j.cej.2011.09.127

[41] Hameed B, Ahmad A. Batch adsorption of methylene blue from aqueous solution by garlic peel, an agricultural waste biomass. Journal of Hazardous Materials. 2009;164(2):870–5. doi: 10.1016/j.jhazmat.2008.08.084 18838221 10.1016/j.jhazmat.2008.08.084

[42] Zhu X, Liu Y, Qian F, Zhou C, Zhang S, Chen J. Preparation of magnetic porous carbon from waste hydrochar by simultaneous activation and magnetization for tetracycline removal. Bioresour Technol. 2014;154:209–14. doi: 10.1016/j.biortech.2013.12.019 24393746 10.1016/j.biortech.2013.12.019

[43] Yuan X, Wu Z, Zhong H, Wang H, Chen X, Leng L, et al Fast removal of tetracycline from wastewater by reduced graphene oxide prepared via microwave-assisted ethylenediamine–N, N’–disuccinic acid induction method. Environmental Science and Pollution Research. 2016;23(18):18657–71. doi: 10.1007/s11356-016-6892-x 27306211 10.1007/s11356-016-6892-x

[44] Qian L, Chen M, Chen B. Competitive adsorption of cadmium and aluminum onto fresh and oxidized biochars during aging processes. J Soils Sediments. 2015;15(5):1130–8. doi: 10.1007/s11368-015-1073-y

