# Supplementary material for: Sorption of tetracycline on biochar derived from rice straw under different temperatures
Source: PLoS One. 2017 Aug 8;12(8):e0182776. doi: 10.1371/journal.pone.0182776 (PMC5549735; doi:10.1371/journal.pone.0182776)
Supplement: S1 Table — (DOC) [file pone.0182776.s001.doc]

**S1 Table.** **Thermodynamic parameters for tetracycline sorption on biochars at different initial concentration of tetracycline (C_0_).**

| **C_0_**  **(mg/L)** | **Biochar** | **Temper**  **-ature**  **(**°C) | **Thermodynamic parameters** | | |
| --- | --- | --- | --- | --- | --- |
|  |  |  | **Δ*G* (kJ/mol)** | **Δ*H* (kJ/mol)** | **Δ*S* (J/(mol·K))** |
| 0.5 | R300 | 15 | -12.50±0.18f* | 37.76±4.95c | 174.06±16.53c |
|  |  | 25 | -13.83±0.10e |  |  |
|  |  | 35 | -16.00±0.09d |  |  |
|  | R500 | 15 | -15.40±0.33d | 50.15±4.28b | 231.73±14.32b |
|  |  | 25 | -21.43±0.32b |  |  |
|  |  | 35 | -19.87±0.14c |  |  |
|  | R700 | 15 | -18.34±0.24c | 62.80±1.43a | 282.14±4.30a |
|  |  | 25 | -21.52±0.03b |  |  |
|  |  | 35 | -23.97±0.16a |  |  |
| 1 | R300 | 15 | -14.17±0.05f | 27.25±2.44c | 143.58±8.97c |
|  |  | 25 | -15.40±0.58ef |  |  |
|  |  | 35 | -17.05±0.15de |  |  |
|  | R500 | 15 | -15.52±1.01ef | 51.94±7.46b | 235.35±27.26b |
|  |  | 25 | -18.80±0.39dc |  |  |
|  |  | 35 | -20.23±1.44bc |  |  |
|  | R700 | 15 | -18.30±0.37d | 77.30±8.18a | 331.10±27.17a |
|  |  | 25 | -20.84±0.11b |  |  |
|  |  | 35 | -24.97±0.20a |  |  |
| 2 | R300 | 15 | -13.53±0.26f | 11.96±3.37c | 88.20±11.37c |
|  |  | 25 | -14.14±0.21fe |  |  |
|  |  | 35 | -15.31±0.25ed |  |  |
|  | R500 | 15 | -16.20±0.63d | 56.94±8.52b | 255.53±26.90b |
|  |  | 25 | -23.55±0.83b |  |  |
|  |  | 35 | -19.87±0.12c |  |  |
|  | R700 | 15 | -18.99±0.63c | 71.13±6.12a | 313.04±19.38a |
|  |  | 25 | -22.25±0.20b |  |  |
|  |  | 35 | -25.24±0.28a |  |  |
| 4 | R300 | 15 | -13.07±0.10e | 11.05±3.63b | 84.38±12.11b |
|  |  | 25 | -14.50±0.23e |  |  |
|  |  | 35 | -14.73±0.15e |  |  |
|  | R500 | 15 | -17.08±0.55d | 76.10±21.38a | 323.43±71.68a |
|  |  | 25 | -20.21±0.72bc |  |  |
|  |  | 35 | -23.55±0.91a |  |  |
|  | R700 | 15 | -18.98±0.63c | 70.55±18.54a | 309.96±61.63a |
|  |  | 25 | -21.25±0.88b |  |  |
|  |  | 35 | -25.22±0.78a |  |  |
| 8 | R300 | 15 | -12.77±0.23f | 26.88±1.72c | 137.65±5.19c |
|  |  | 25 | -14.13±0.16fe |  |  |
|  |  | 35 | -15.52±0.12ed |  |  |
|  | R500 | 15 | -16.08±0.70ed | 54.66±9.63b | 246.75±31.18b |
|  |  | 25 | -19.57±0.94c |  |  |
|  |  | 35 | -20.97±0.68bc |  |  |
|  | R700 | 15 | -17.27±0.59d | 91.27±13.77a | 378.80±49.36a |
|  |  | 25 | -22.94±0.85ab |  |  |
|  |  | 35 | -24.77±1.59a |  |  |
| 16 | R300 | 15 | -12.29±0.27d | 12.11±6.11c | 84.83±28.12c |
|  |  | 25 | -13.23±0.17d |  |  |
|  |  | 35 | -13.98±0.42d |  |  |
|  | R500 | 15 | -15.92±0.22c | 55.34±4.50b | 245.14±15.75b |
|  |  | 25 | -16.30±0.46c |  |  |
|  |  | 35 | -20.92±0.46b |  |  |
|  | R700 | 15 | -17.55±0.46c | 75.70±12.93a | 324.12±45.04a |
|  |  | 25 | -21.10±0.83b |  |  |
|  |  | 35 | -24.02±1.30a |  |  |
| 24 | R300 | 15 | -11.41±0.24e | 13.42±1.65c | 86.38±6.20b |
|  |  | 25 | -12.42±0.17e |  |  |
|  |  | 35 | -13.13±0.34ed |  |  |
|  | R500 | 15 | -14.49±0.22d | 65.77±3.51a | 278.23±13.04a |
|  |  | 25 | -16.86±0.49c |  |  |
|  |  | 35 | -20.07±0.46b |  |  |
|  | R700 | 15 | -17.81±0.57c | 52.85±5.52b | 245.65±18.11a |
|  |  | 25 | -20.53±1.27b |  |  |
|  |  | 35 | -22.71±0.82a |  |  |
| 32 | R300 | 15 | -11.17±0.18g | 9.98±5.62c | 81.85±16.98c |
|  |  | 25 | -11.99±0.18fg |  |  |
|  |  | 35 | -12.63±0.42f |  |  |
|  | R500 | 15 | -14.47±0.24e | 44.64±11.67b | 204.80±39.87b |
|  |  | 25 | -16.09±0.42d |  |  |
|  |  | 35 | -18.59±0.55c |  |  |
|  | R700 | 15 | -16.16±0.44d | 75.70±8.46a | 319.81±30.30a |
|  |  | 25 | -20.11±0.46b |  |  |
|  |  | 35 | -22.52±1.07a |  |  |

* The letters indicate significant differences for the results under same initial concentration of tetracycline at a level of p < 0.05 (Tukey test).
